# Phylogenetic analysis of human *Chlamydia pneumoniae* strains reveals a distinct Australian indigenous clade that predates European exploration of the continent

**DOI:** 10.1186/s12864-015-2281-y

**Published:** 2015-12-22

**Authors:** Eileen Roulis, Nathan Bachmann, Michael Humphrys, Garry Myers, Wilhelmina Huston, Adam Polkinghorne, Peter Timms

**Affiliations:** Institute of Health and Biomedical Innovation, Queensland University of Technology, Kelvin Grove, QLD Australia; Centre for Animal Health Innovation, Faculty of Science, Health, Education & Engineering, University of the Sunshine Coast, Sippy Downs, QLD Australia; Institute for Genomic Sciences, University of Maryland, Baltimore, MD USA; i3 Institute, University of Technology, Sydney, NSW Australia

**Keywords:** Chlamydia pneumoniae, Whole genome sequencing, Phylogenomics, Polymorphisms, Inclusion proteins, Polymorphic membrane protein, Inosine-monophosphate dehydrogenase, Recombination, Infection

## Abstract

**Background:**

The obligate intracellular bacterium *Chlamydia pneumoniae* is a common respiratory pathogen, which has been found in a range of hosts including humans, marsupials and amphibians. Whole genome comparisons of human *C. pneumoniae* have previously highlighted a highly conserved nucleotide sequence, with minor but key polymorphisms and additional coding capacity when human and animal strains are compared.

**Results:**

In this study, we sequenced three Australian human *C. pneumoniae* strains, two of which were isolated from patients in remote indigenous communities, and compared them to all available *C. pneumoniae* genomes. Our study demonstrated a phylogenetically distinct human *C. pneumoniae* clade containing the two indigenous Australian strains, with estimates that the most recent common ancestor of these strains predates the arrival of European settlers to Australia. We describe several polymorphisms characteristic to these strains, some of which are similar in sequence to animal *C. pneumoniae* strains, as well as evidence to suggest that several recombination events have shaped these distinct strains.

**Conclusions:**

Our study reveals a greater sequence diversity amongst both human and animal *C. pneumoniae* strains, and suggests that a wider range of strains may be circulating in the human population than current sampling indicates.

## Background

*Chlamydia pneumoniae* is an obligate intracellular bacterium and member of the *Chlamydiaceae*, a family of pathogens of higher eukaryotes with a distinct biphasic development cycle [1]. Whilst *C. pneumoniae* is primarily recognised as an aetiological agent of community acquired pneumonia and other respiratory diseases in humans [2], it has a broad host range encompassing both warm [3–5] and cold blooded animals [6, 7]. Members of the *Chlamydiaceae* are characterised by their compact genomes and highly conserved gene content [8]. *C. pneumoniae* has the greatest coding capacity of the *Chlamydiaceae*, with animal strains of *C. pneumoniae* having between 20Kbp (animal versus human *C. pneumoniae*) to almost 200Kbp (animal *C. pneumoniae* versus *C. trachomatis* serovar D) [9, 10] of extra nucleotide sequence. The additional coding capacity of *C. pneumoniae* is predominantly accounted for by the expansion of the polymorphic membrane protein (*pmp*) and inclusion membrane protein (*inc*) gene families [10–12], both of which are involved in the formation and maintenance of the chlamydial inclusion body, modulation of the host cell response [12, 13], as well as a large number of species-specific metabolic and hypothetical protein genes [9, 10, 14].

In addition to its description as a cause of human respiratory disease, *C. pneumoniae* has been implicated in a variety of human pathologies, including cardiovascular disease, Alzheimer’s disease, ischaemic stroke, asthma and lung cancer [15–18]. Until recently, the majority of fully sequenced *C. pneumoniae* whole genomes were from strains that were isolated from respiratory pathologies [10, 19, 20], and demonstrated highly conserved nucleotide sequence content and gene order. Recently, several genomes from respiratory and cardiovascular strains were reported, as were whole genome sequences from atherosclerotic and Alzheimer’s *C. pneumoniae* strains, which allowed for comparison of strains isolated from different diseases, and demonstrated that only minor genetic differences were found between these strains [9, 21, 22].

A previous study examining the genetic diversity between human and animal *C. pneumoniae* suggested that a genetically distinct strain of human *C. pneumoniae* was present and circulating within Australian indigenous communities [23]. PCR analysis of a small number of selected target genes was performed on two respiratory strains isolated from Indigenous Australian patients in geographically separate regions [24, 25] and these were shown to have nucleotide sequence, that in some instances, placed these strains phylogenetically closer to animal strains of *C. pneumoniae* than those circulating in human populations in Australia and worldwide [23].

To further explore the genetic diversity of Australian human *C. pneumoniae* strains, we genome sequenced and performed comparative genomic and phylogenetic analyses of two human Australian indigenous *C. pneumoniae* strains and a third strain from an Australian Caucasian patient. In doing so, we (i) demonstrate that the indigenous Australian human strains form a separate clade branching earlier than other human *C. pneumoniae* strains; (ii) identify genetic markers unique to Australian indigenous and non-indigenous strains; and (iii) reveal evidence of limited recombination within *C. pneumoniae* strains from the greater human *C. pneumoniae* clade.

## Results

### Phylogenetic relationships in human *C. pneumoniae* reveal a distinct Australian indigenous clade predating European exploration of the continent

*C. pneumoniae* strains SH511, 1979 [24, 25] and WA97001 [26] were sequenced following capture of *C. pneumoniae* DNA using a set of species-specific SureSelect^XT^ RNA probes [27–29]. Sequencing reads of *C. pneumoniae* WA97001, SH511 and 1797 were mapped to the reference genome, *C. pneumoniae* AR39, to check the efficacy of the SureSelect^XT^ DNA captures. The genome of SH511 had the highest mean read depth of 1944×, followed by 1979, which had an average read depth of 1887×. The SH511 and 1979 assembled into 10 contigs and 31 contigs, respectively. In contrast, *C. pneumoniae* WA97001 genome had a significantly lower read depth of 15× and assembled into 104 contigs.

In order to determine the evolutionary and phylogenetic relationships between the Australian *C. pneumoniae* strains and those previously published, Bayesian and coalescent estimation methods were used to construct phylogenetic trees based on whole genome alignments of all human *C. pneumoniae* strains and the three published animal *C. pneumoniae* strains.

**Fig. 1:**
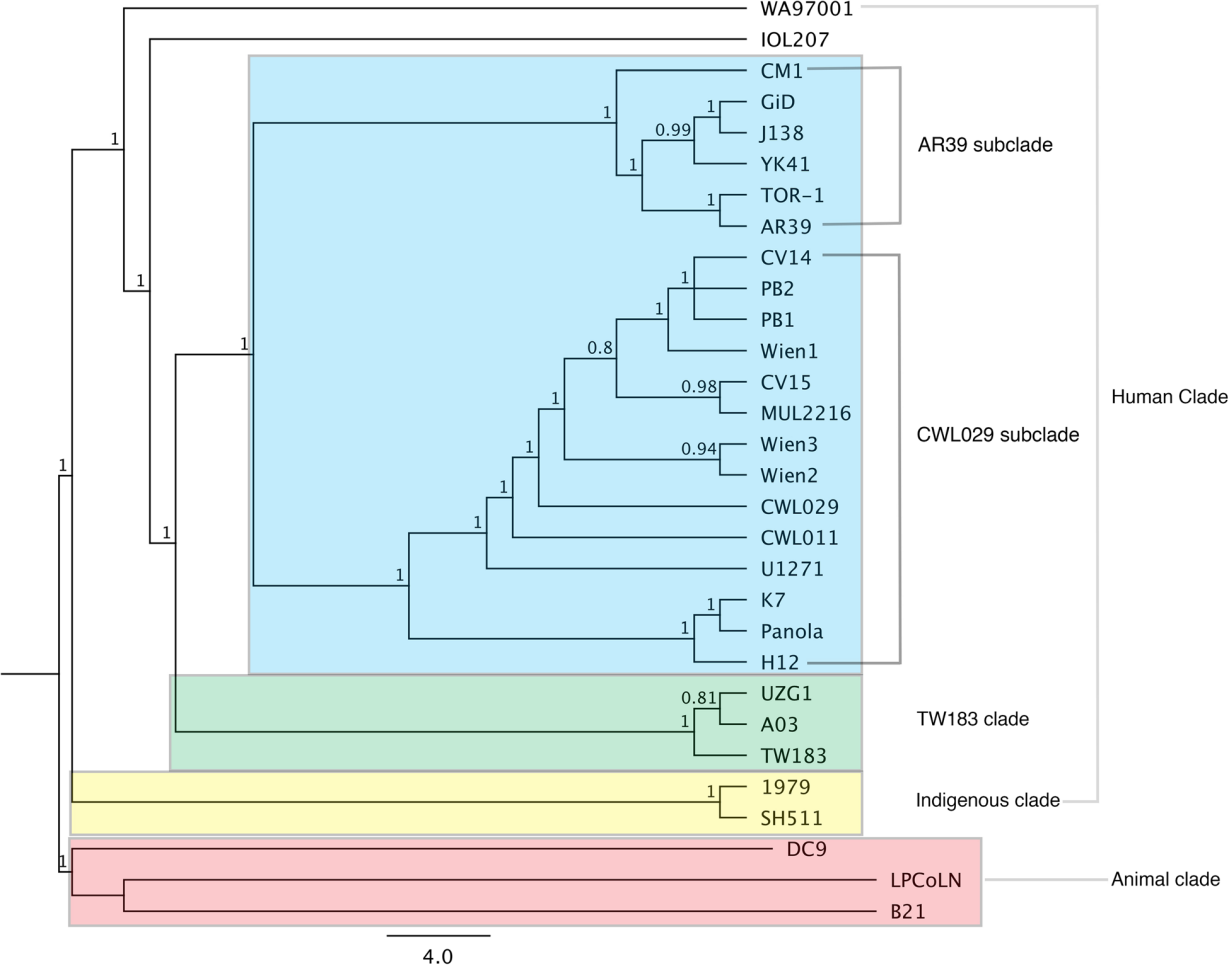
*Chlamydia pneumoniae* whole genome phylogeny constructed using MrBayes. Posterior probabilities >0.75 shown. Animal and human *C. pneumoniae* strains form the two major clades, with four distinct clades within the human *C. pneumoniae* tree

Percentage pairwise identities between indigenous and non-indigenous strains ranged from 98.4 to 98.8 %, whilst non-indigenous strains were 99.0 % or greater. Percentage identities of all strains used in the MrBayes analysis are outlined in Table 1. The resulting phylogenetic tree as represented in Fig. 1, demonstrates a clear demarcation of animal and human clades. The majority of non-indigenous human strains cluster into two clades: a large single clade that contains the AR39 and CWL029 subclades, and the smaller TW183 clade [22]. Interestingly, the two Australian indigenous *C. pneumoniae* strains, SH511 and 1979, formed their own clade which branched deepest from the main human *C. pneumoniae* grouping, but was also considerably distant to the animal *C. pneumoniae* clade. The Australian caucasian strain WA97001 and IOL207 (a strain isolated from a case of acute conjunctivitis) [30] formed their own separate branches in the main human *C. pneumoniae* clade.

**Table 1:** Percentage nucleotide pairwise identities of all *C. pneumoniae* strains

	CM1	GiD	TOR-1	AR39	YK41	J138	CV14	PB2	PB1	CWL011	Wien3	CWL029	U1271	CV15	Wien1	K7	Wien2	MUL2216	Panola	H12	UZG1	TW183	A03	WA97001	1979	SH511	IOL207	DC9	LPCoLN	B21
CM1		100	100	100	99.9	99.7	99.7	99.7	99.7	99.8	99.8	99.6	99.8	99.7	99.7	99.8	99.8	99.8	99.8	99.4	99.6	99.6	99.5	99.7	98.6	98.7	99.2	97.9	94.6	94.6
GiD	100		100	100	99.9	99.7	99.7	99.7	99.7	99.8	99.8	99.6	99.8	99.7	99.7	99.8	99.8	99.8	99.8	99.4	99.6	99.6	99.5	99.7	98.6	98.7	99.2	97.9	94.6	94.6
TOR-1	100	100		100	99.9	99.7	99.7	99.7	99.7	99.8	99.8	99.6	99.8	99.7	99.7	99.8	99.8	99.8	99.8	99.4	99.6	99.6	99.5	99.7	98.6	98.7	99.2	97.9	94.6	94.6
AR39	100	100	100		99.9	99.7	99.7	99.7	99.7	99.8	99.8	99.6	99.8	99.7	99.7	99.8	99.8	99.8	99.8	99.4	99.6	99.6	99.5	99.7	98.6	98.7	99.2	97.9	94.6	94.6
YK41	99.9	99.9	99.9	99.9		99.6	99.7	99.7	99.7	99.7	99.7	99.6	99.7	99.7	99.7	99.7	99.7	99.7	99.7	99.4	99.5	99.5	99.5	99.7	98.6	98.6	99.2	97.9	94.6	94.6
J138	99.7	99.7	99.7	99.7	99.6		99.7	99.7	99.7	99.7	99.7	99.6	99.7	99.7	99.7	99.7	99.7	99.7	99.7	99.4	99.5	99.5	99.5	99.5	98.7	98.7	99.2	97.9	94.8	94.8
CV14	99.7	99.7	99.7	99.7	99.7	99.7		100	100	100	100	99.8	100	100	100	100	100	100	100	99.6	99.7	99.7	99.6	99.5	98.7	98.7	99.4	98	94.7	94.7
PB2	99.7	99.7	99.7	99.7	99.7	99.7	100		100	100	100	99.8	100	100	100	99.9	99.9	99.9	99.9	99.6	99.7	99.7	99.6	99.5	98.7	98.7	99.4	98	94.7	94.7
PB1	99.7	99.7	99.7	99.7	99.7	99.7	100	100		100	100	99.8	100	100	100	100	100	100	99.9	99.6	99.7	99.7	99.6	99.5	98.7	98.7	99.4	98	94.7	94.7
CWL011	99.8	99.8	99.8	99.8	99.7	99.7	100	100	100		100	99.9	100	100	100	100	100	100	100	99.7	99.7	99.7	99.6	99.5	98.7	98.8	99.4	98	94.7	94.7
Wein3	99.8	99.8	99.8	99.8	99.7	99.7	100	100	100	100		99.9	100	100	100	100	100	100	100	99.7	99.7	99.7	99.6	99.5	98.7	98.8	99.4	98	94.7	94.7
CWL029	99.6	99.6	99.6	99.6	99.6	99.6	99.8	99.8	99.8	99.9	99.9		99.9	99.8	99.8	99.9	99.9	99.9	99.9	99.5	99.6	99.6	99.8	99.4	98.9	98.9	99.3	97.9	94.6	94.6
U1271	99.8	99.8	99.8	99.8	99.7	99.7	100	100	100	100	100	99.9		100	100	100	100	100	100	99.7	99.7	99.7	99.6	99.5	98.7	98.8	99.4	98	94.7	94.7
CV15	99.7	99.7	99.7	99.7	99.7	99.7	100	100	100	100	100	99.8	100		100	100	100	100	100	99.6	99.7	99.7	99.6	99.5	98.7	98.7	99.4	98	94.7	94.7
Wein1	99.7	99.7	99.7	99.7	99.7	99.7	100	100	100	100	100	99.8	100	100		100	100	100	100	99.6	99.7	99.7	99.6	99.5	98.7	98.7	99.4	98	94.7	94.7
K7	99.8	99.8	99.8	99.8	99.7	99.7	100	99.9	100	100	100	99.9	100	100	100		100	100	100	99.7	99.7	99.7	99.6	99.5	98.7	98.8	99.4	98	94.7	94.7
Wein2	99.8	99.8	99.8	99.8	99.7	99.7	100	99.9	100	100	100	99.9	100	100	100	100		100	100	99.7	99.7	99.7	99.6	99.5	98.7	98.8	99.4	98	94.7	94.7
MUL2216	99.8	99.8	99.8	99.8	99.7	99.7	100	99.9	100	100	100	99.9	100	100	100	100	100		100	99.7	99.7	99.7	99.6	99.5	98.7	98.8	99.4	98	94.7	94.7
Panola	99.8	99.8	99.8	99.8	99.7	99.7	100	99.9	99.9	100	100	99.9	100	100	100	100	100	100		99.7	99.7	99.7	99.6	99.5	98.7	98.8	99.4	98	94.7	94.7
H12	99.4	99.4	99.4	99.4	99.4	99.4	99.6	99.6	99.6	99.7	99.7	99.5	99.7	99.6	99.6	99.7	99.7	99.7	99.7		99.4	99.4	99.3	99.2	98.4	98.4	99.1	97.8	94.4	94.4
UZG1	99.6	99.6	99.6	99.6	99.5	99.5	99.7	99.7	99.7	99.7	99.7	99.6	99.7	99.7	99.7	99.7	99.7	99.7	99.7	99.4		100	99.6	99.3	98.8	98.8	99.2	98.2	94.5	94.5
TW183	99.6	99.6	99.6	99.6	99.5	99.5	99.7	99.7	99.7	99.7	99.7	99.6	99.7	99.7	99.7	99.7	99.7	99.7	99.7	99.4	100		99.6	99.3	98.8	98.8	99.2	98.2	94.5	94.5
A03	99.5	99.5	99.5	99.5	99.5	99.5	99.6	99.6	99.6	99.6	99.6	99.8	99.6	99.6	99.6	99.6	99.6	99.6	99.6	99.3	99.6	99.6		99.3	98.9	98.9	99.1	97.9	94.5	94.4
WA97001	99.7	99.7	99.7	99.7	99.7	99.5	99.5	99.5	99.5	99.5	99.5	99.4	99.5	99.5	99.5	99.5	99.5	99.5	99.5	99.2	99.3	99.3	99.3		98.8	98.8	99	97.8	94.5	94.5
1979	98.6	98.6	98.6	98.6	98.6	98.7	98.7	98.7	98.7	98.7	98.7	98.9	98.7	98.7	98.7	98.7	98.7	98.7	98.7	98.4	98.8	98.8	98.9	98.8		99.7	98.4	97.5	94.2	94.2
SH511	98.7	98.7	98.7	98.7	98.6	98.7	98.7	98.7	98.7	98.8	98.8	98.9	98.8	98.7	98.7	98.8	98.8	98.8	98.8	98.4	98.8	98.8	98.9	98.8	99.7		98.4	97.5	94.3	94.3
IOL207	99.2	99.2	99.2	99.2	99.2	99.2	99.4	99.4	99.4	99.4	99.4	99.3	99.4	99.4	99.4	99.4	99.4	99.4	99.4	99.1	99.2	99.2	99.1	99	98.4	98.4		97.6	94.3	94.3
DC9	97.9	97.9	97.9	97.9	97.9	97.9	98	98	98	98	98	97.9	98	98	98	98	98	98	98	97.8	98.2	98.2	97.9	97.8	97.5	97.5	97.6		93.6	93.6
LPCoLN	94.6	94.6	94.6	94.6	94.6	94.8	94.7	94.7	94.7	94.7	94.7	94.6	94.7	94.7	94.7	94.7	94.7	94.7	94.7	94.4	94.5	94.5	94.5	94.5	94.2	94.3	94.3	93.6		100
B21	94.6	94.6	94.6	94.6	94.6	94.8	94.7	94.7	94.7	94.7	94.7	94.6	94.7	94.7	94.7	94.7	94.7	94.7	94.7	94.4	94.5	94.5	94.4	94.5	94.2	94.3	94.3	93.6	100

To investigate the evolutionary relationships of these deep-branching Australian indigenous human strains further, we determined the date of the most recent common ancestor (MRCA) of the indigenous Australian *C. pneumoniae* strains by using BEAST [31] and ClonalFrame [32] coalescent estimation methods. BEAST analysis of indigenous and non-indigenous *C. pneumoniae* strains reveals an MRCA for indigenous strains at 1028, with a 95 % credibility interval between 996 and 1062 years. The mean substitution rate was determined to be 4.64 × 10^−4^ substitutions per site, per year. ClonalFrame analysis of indigenous and non-indigenous *C. pneumoniae* strains reveals a MRCA of 1425 for the indigenous strains, with a mean substitution rate of 2.36 × 10^−5^ per site per year. Though there are minor differences in the predicted MRCA and substitution rates between the two programs, which can be accounted for by the difference in their calculation methods [33], their estimates support similar evolutionary timelines and dates.

### Identification of genetic markers that distinguish Australian indigenous strains from non-indigenous and animal *C. pneumoniae* strains

Using a PCR-based sequencing approach, we previously identified a series of potential genetic markers that could be used to distinguish Caucasian *C. pneumoniae* strains of different origins [23]. In the current study, fine-detailed genomic comparisons identified a series of novel genetic markers unique to the Australian indigenous strains, as well as unexpected sequence diversity in the DC9, WA97001 and IOL207 strains, which support their distinct phylogenetic positions in the *C. pneumoniae* tree.

**Fig. 2:**
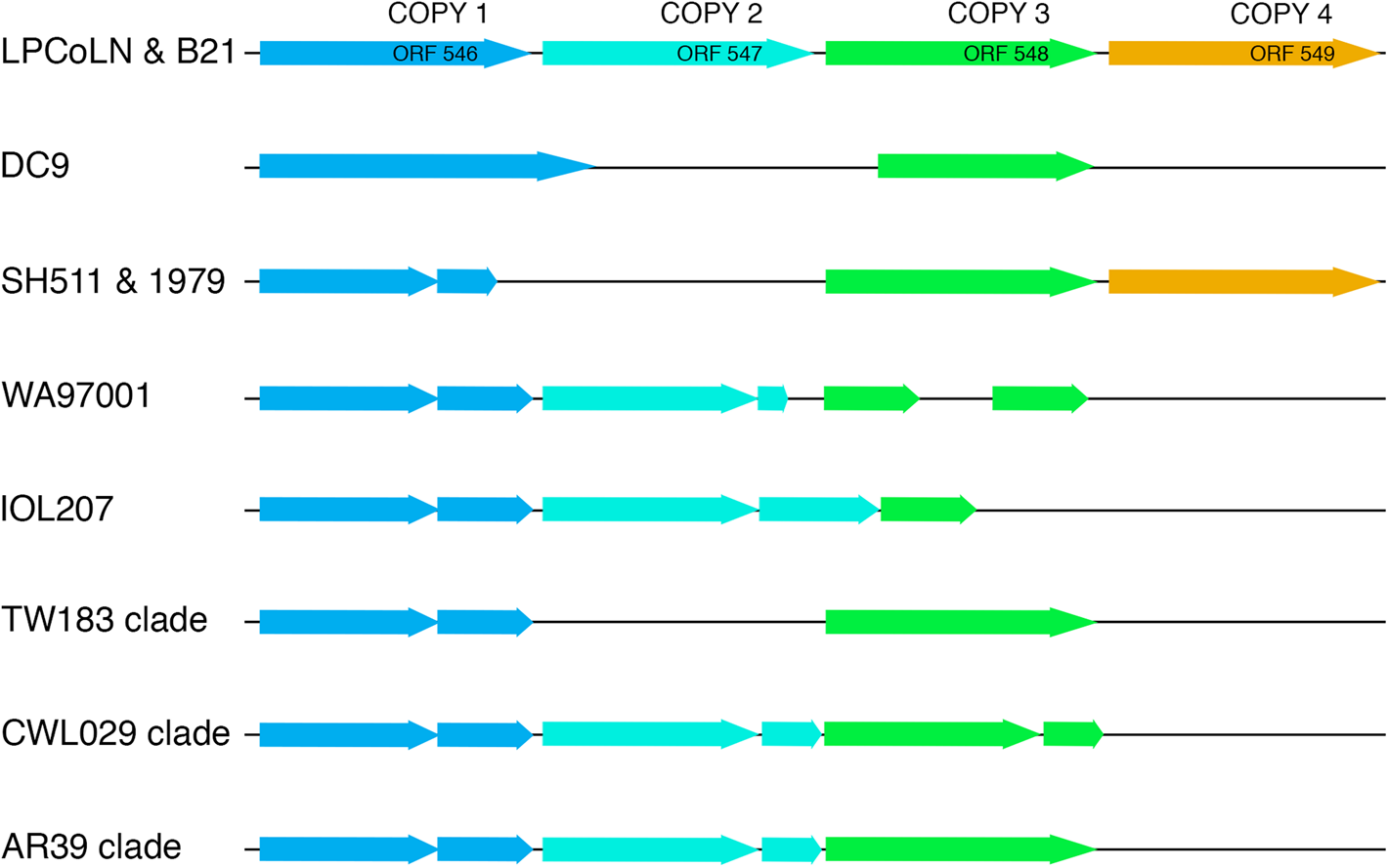
The *IncA* gene expansion and recombination locus spanning homologs of CPK_ORF546 through 549 (LPCoLN locus numbering). Human and frog *C. pneumoniae* strains encode for either two or three copies with various levels of fragmentation between strains. Different clades within human *C. pneumoniae* encode for identical sequence length across this locus. The Australian indigenous strains are the only known human *C. pneumoniae* strains to encode for a CPK_ORF00549 homolog

One of the most significant regions of genetic variation identified is located around four full-length *IncA* genes annotated in koala strain LPCoLN (CPK_ORF00546 to CPKORF00549 [9]); the differences of which support our phylogenetic results. The most notable finding in this region for the three Australian strains was the observation that the Australian indigenous strains contain a full-length homolog of CPK_ORF00549 sharing 99.4 % nucleotide pairwise identity to the koala homolog (Fig. 2). The presence of this gene in strains SH511 and 1979, and its significant sequence identity to the koala/bandicoot homolog supports the branching of the Australian indigenous clade earliest in the greater human *C. pneumoniae* phylogeny. Conversely, the Australian indigenous strains do not have a copy of CPK_ORF00547. This locus is also absent in the frog (DC9) strain and all strains within the TW183 clade, but is found in fragmented forms in all other human strains. Gene copy numbers and fragmentation with respect to the koala LPCoLN strain is represented in Fig. 2.

**Fig. 3:**
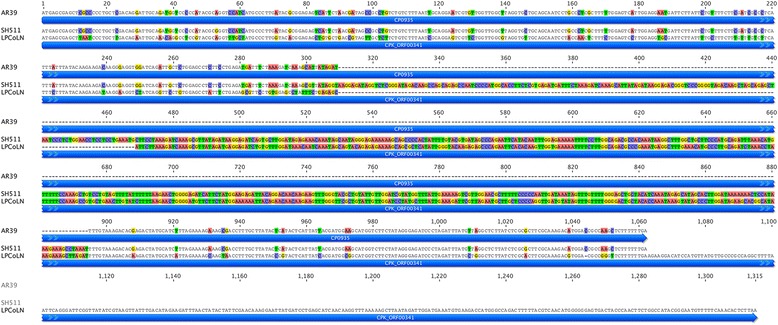
A large sequence insertion is specific to indigenous strains SH511 and 1979, within a putative *IncA* gene homologous to CPK_ORF341. This insertion encodes an almost full-length *IncA* homolog similar to that in the koala and bandicoot strains. Sequences at this locus for SH511 and 1979 are identical, and are shown compared to human strain AR39 and koala strain LPCoLN

Another genetic marker unique to the Australian indigenous *C. pneumoniae* strains SH511 and 1979, was the presence of a 159 bp insertion in the gene homologous to koala CPK_ORF0341 (585 bp insertion compared to the AR39 homolog). Translation of the open reading frame suggests that this is a putative *IncA* gene which is full length in the koala strain. However, this gene is slightly truncated by 84 amino acids in indigenous strains (354 amino acids in length) and is only 154 amino acids in length in all other strains, including frog DC9 - due to a single nucleotide insertion 3’ which results in a frame shift (Fig. 3). Again, the large, strain-specific insertion and its sequence similarity to the koala homolog, supports the earliest branching of the Australian indigenous strains in the major human *C. pneumoniae* clade.

**Fig. 4:**
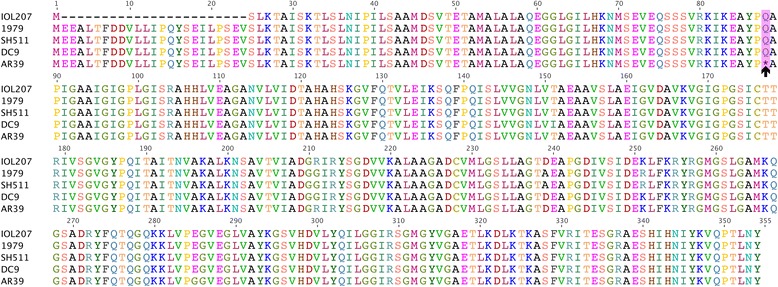
A single nucleotide transition in strains SH511, 1979, DC9 and IOL207 results in a full-length *guaB* gene, compared to fragmented genes in other human *C. pneumoniae* represented by AR39. The amino acid residue change at position 88 in strains IOL207, 1979, SH511 and DC9 is highlighted in the pink box, whilst the black arrow below the AR39 sequence indicates the *guaB* stop codon which is present in all other human *C. pneumoniae* strains. The IOL207 homolog is N-terminal truncated by 23 amino acids

Sequence polymorphism has been described in the *guaB/A-add* operon in human and animal *C. pneumoniae* strains, with previous studies detailing that human *C. pneumoniae* strains encode fragmented inosine-5-monophosphate dehydrogenase (*guaB*) genes [9]. In this study, we found that like the DC9 frog strain, the Australian indigenous strains and strain IOL207 encode for a full length, intact *guaB* gene. By comparison, all other human strains have a T/C transition at nucleotide position 262, which results in a stop codon (Fig. 4). Varied levels of sequence decay are evident in the Australian strains for GMP synthase (*guaA*) and adenosine deaminase (*add*). Deletions in both the *guaA* and *add* homologs of WA97001 result in truncations of these genes with loss of functional domains, whilst the Australian indigenous strains exhibit extensive sequence decay at this locus, resulting in the absence of *guaA-add* and the downstream hypothetical protein. Interestingly, whilst the entire *guaA/B-add* operon is absent in both koala and bandicoot strains, these genes are present in the frog strain DC9.

**Fig. 5:**
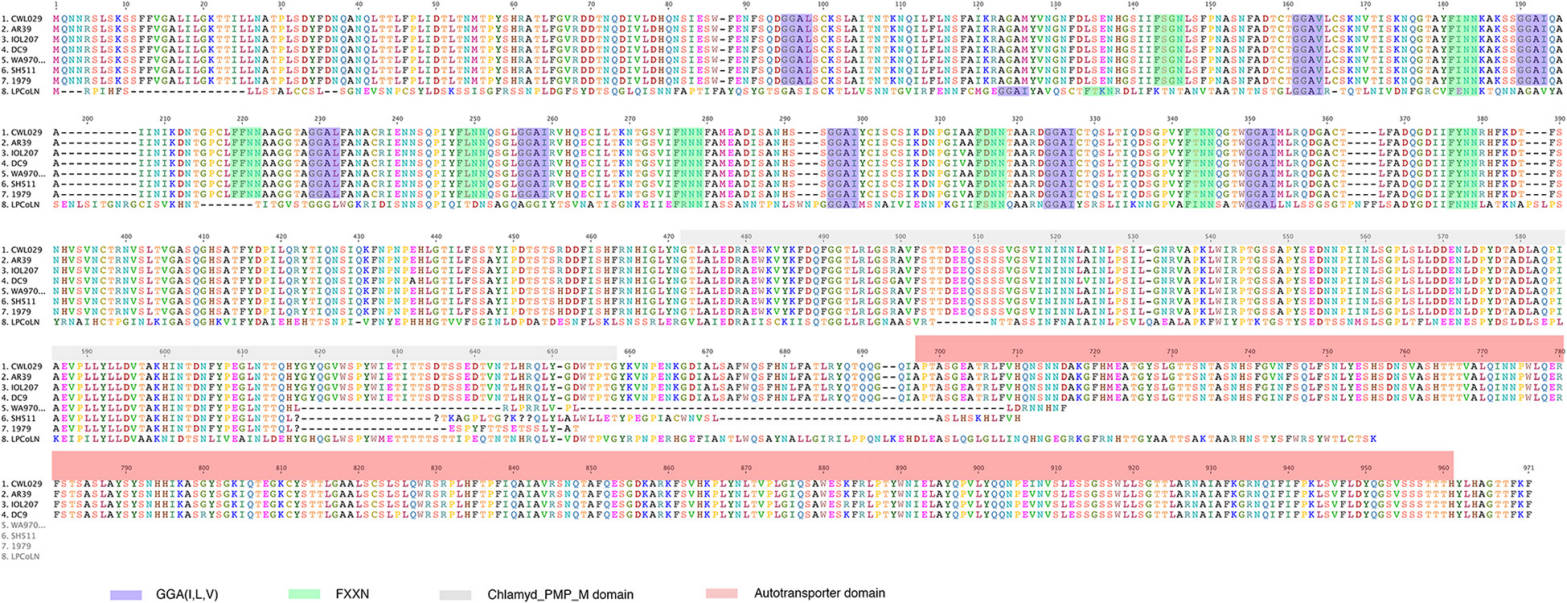
*pmpE/F4* displays significant sequence polymorphism and decay in Australian *C. pneumoniae* strains SH511, 1979 and WA97001, resulting in truncated homologs of this protein. The frog DC9 homolog is similar in sequence to human *C. pnuemoniae* strains, unlike the koala and bandicoot strains which are highly polymorphic at this locus. The GGA(I,L,V) - FXXN amino acid repeat motifs characteristic to the polymorphic membrane protein gene family are highlighted, whilst sequence for the C-terminal autotransporter domain is clearly absent in SH511, 1979, WA97001 and LPCoLN strains

Various sequence polymorphisms are evident in the Australian *C. pneumoniae* strains for the *pmpE/F4* gene. Both indigenous Australian strains 1979 and SH511 are truncated as the result of several deletions, whilst a single nucleotide insertion in WA97001 results in a frameshift causing truncation of this gene. This results in the loss of the C-terminal autotransporter domain for all three strains - however the mid-gene region encoding for nine FXXN and eight GGA(I,L,V) amino acid motifs are highly conserved across all the human *C. pneumoniae* strains (Fig. 5). Additionally, whilst both the koala and bandicoot homologs of this gene display extensive sequence polymorphism, the DC9 frog homolog is highly similar in sequence to the non-indigenous human *pmpE/F4* and encodes for the full-length protein.

### Australian indigenous strains demonstrate characteristic recombination profiles with only a few instances shared with non-indigenous strains

**Fig. 6:**
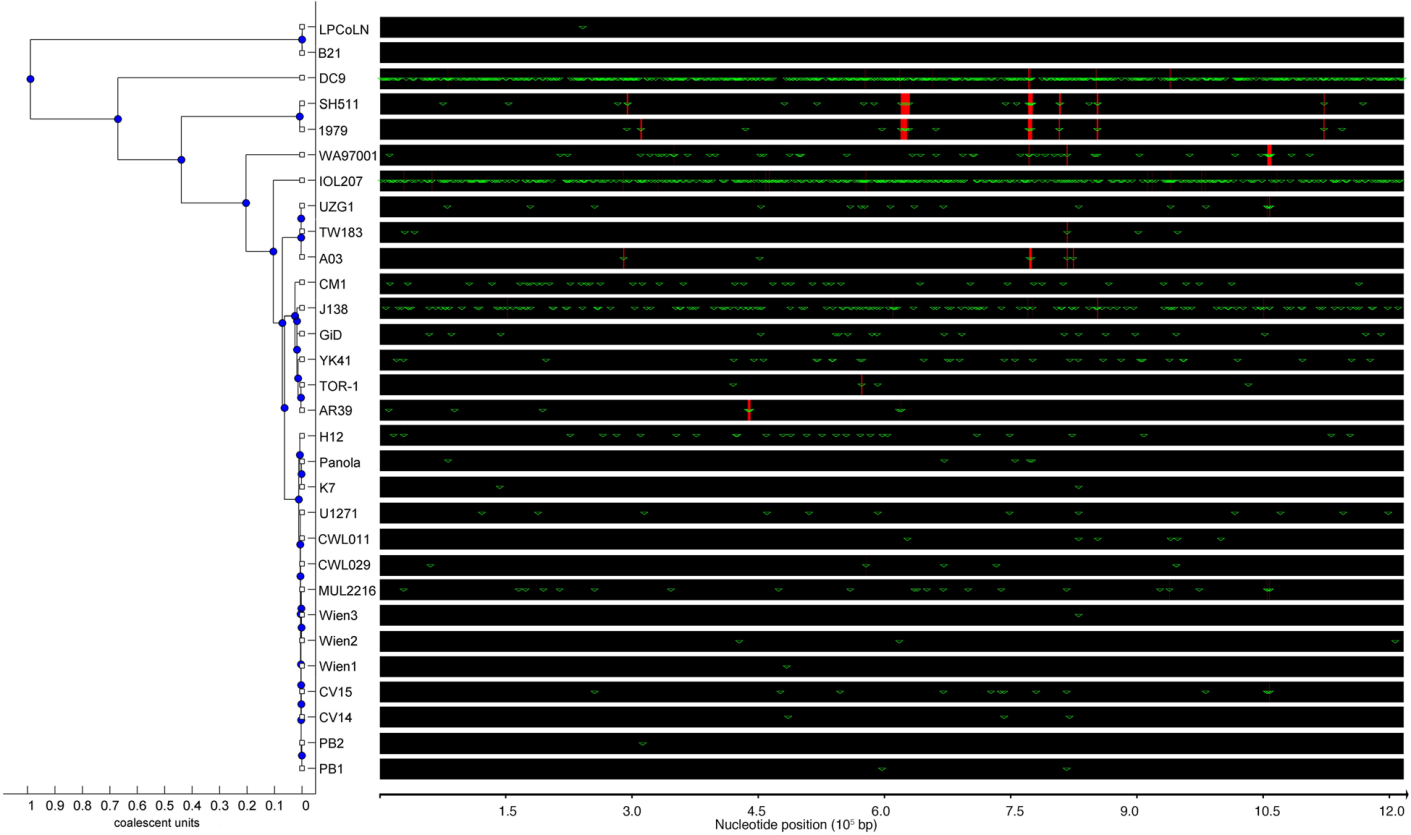
Whole genome recombination mappings as predicted by ClonalFrame coalescent methods. Red bars represent recombination events and green ticks represent mutations. Strains SH511 and 1979 share almost identical recombination profiles, with non-indigenous human *C. pneumoniae* and the DC9 frog strain sharing recombination events at discrete loci. The predicted whole genome phylogeny based on recombination and mutation events is consistent with the groupings demonstrated using BEAST and MrBayes prediction methods

In addition to estimation of the MRCA and mean substitution rate, ClonalFrame was used to determine the recombination profiles and any shared recombination loci in *C. pneumoniae*. Our study found that the Australian indigenous strains SH511 and 1979 had a distinct and almost identical recombination and nucleotide substitution profile, with only a single difference in recombination locus between the two: SH511 between 296,000 and 298,000 bp and 1979 between 310,000 and 316,000 bp. Additionally, SH511 and 1979 share a strongly supported recombination event with the atherosclerosis strain A03 and to a lesser extent with Australian non-indigenous strain WA97001 between 778,000 and 784,000, which encompasses hypothetical protein and putative *IncA* genes. In comparing recombination profiles across the non-indigenous *C. pneumoniae* strains, the Australian WA97001 strain shares a single strong recombination event with A03 and TW183 between 823,600 and 827,100 bp, which encompasses putative *IncA* genes. Several nucleotide substitution events are shared amongst the various *C. pneumoniae* strains, though the highest number of nucleotide substitutions occur in strains J138, IOL207 and DC9 (Fig. 6). A Phi test for recombination was performed on the *C. pneumoniae* whole genome alignment using SplitsTree4 [34], which found a total of 16,329 informative sites and statistically significant evidence of recombination (*p* = 5.538 × 10^−4^).

## Discussion

*C. pneumoniae* has been described as an ancient pathogen, with the broadest host range of any member of the *Chlamydiaceae* [35]. Comparative whole genome studies examining the differences between human respiratory [20], non-respiratory [9, 21, 22, 36] and animal *C. pneumoniae* strains [9] all demonstrate a highly conserved core genome with subtle strain-specific differences. We previously characterized some of these subtle differences using a PCR/sequencing approach and revealed that the two human Australian indigenous human strains sequenced in this study shared genetic markers with the koala LPCoLN strain [9] for some genes and away from other human non-Australian indigenous strains [23]. To further explore the relationship of Australian indigenous and non-indigenous human strains, in the current study, we obtained whole genome sequences for three Australian respiratory strains (SH511, 1979 and WA97001) and performed comparative analyses to further understand their relationship to other previously characterized human and animal *C. pneumoniae* strains.

Using a variety of phylogenomic tools, our analysis suggests that the Australian *C. pneumoniae* indigenous strains form a phylogenetically distinct clade away from all other human *C. pneumoniae* strains sequenced to date. This is substantiated by unique sequence polymorphisms and recombination profiles associated with the Australian indigenous strains. In contrast to previous phylogenies constructed using sequenced PCR fragments, which alternately placed the Australian indigenous strains within either the human or animal branches of the tree [23], the use of whole genome sequences gives a more accurate description of the position of these strains within the greater *C. pneumoniae* evolutionary tree. Fine-detailed genomic comparisons also revealed several novel genetic markers in Australian indigenous human *C. pneumoniae* strains, beyond those previously identified in previous PCR-based studies [23].

The Australian indigenous strains demonstrate a copy number incongruity within the CPK_ORF00546 to CPK_ORF00549 *IncA* gene family. This gene family expansion was first described in the koala LPCoLN strain [9] with human *C. pneumoniae* strains exhibiting variable levels of gene fragmentation and gene loss at this locus. The Australian indigenous strains are unique in that they specifically encode a homolog to CPK_ORF00549: to date, SH511 and 1979 are the only human *C. pnuemoniae* strains that encode for this homolog. Previous studies have shown that *C. pneumoniae* encodes a far larger number of *IncA* and putative *IncA* proteins compared to other *Chlamydiae* [11, 12], many of which are species-specific. Strong recombination signals were also detected within several human *C. pneumoniae* strains at loci encoding *IncA* proteins, which suggests that recombination may account for the expanded number of *IncA* proteins in *C. pneumoniae*.

One of the more subtle genetic differences observed between the strains analysed was the maintenance of a partial purine biosynthesis pathway encoded by *guaA/B-add* [10]. Previous studies demonstrated that the *guaB* gene is fragmented in human *C. pneumoniae* strains [14], however in this study we demonstrate that strains DC9, SH511 and 1979 encode for an intact *guaB* gene. Given that the Australian indigenous strains do not encode *guaA-add*, it is likely that the sequence for *guaB* was a recent acquisition from a strain most similar to DC9. Interestingly, in contrast to the koala and bandicoot strains where the entire *guaA/B-add* operon is absent [9, 37], the frog DC9 strain encodes *guaA/B*-*add* genes, with >99.5 % nucleotide pairwise identity to all human *C. pneumoniae* strains, with the exception of the three Australian strains. Studies in both *C. psittaci* and *Chlamydia caviae* have found evidence for horizontal gene transfer of the *guaA/B-add* operon between different chlamydial strains and species [33, 38], lending further support for the recent acquisition of *guaB* by the Australian indigenous strains. Whilst it is unclear what effect the presence or absence of *guaA/B-add* has on the growth and virulence of human and animal *C. pneumoniae* strains, a previous study examining the effect of mutations in the *Chlamydia muridarum* plasticity zone suggest that 5’ point mutations of *guaB* and *add* result in attenuated virulence in vivo, whilst *guaA/B-add* mutations do not affect the growth characteristics of these strains in vitro [39]. These observations are similar to those reported for the growth and virulence of *Borrelia burgdorferi* and *Francisella tularensis guaA/B* +/− strains in vitro and in vivo [40, 41].

In order to further explore the evolutionary relationships of the Australian indigenous *C. pneumoniae* strains, BEAST and ClonalFrame analyses predicted that these strains had an MRCA of 1028 and 1425, respectively. Both of these estimations pre-date the known colonization of the Australian continent by Europeans by several hundred years, but are virtually identical to the previously estimated MRCA for strains within the non-indigenous clade at 1151 +/− 20 years [21].

**Fig. 7:**
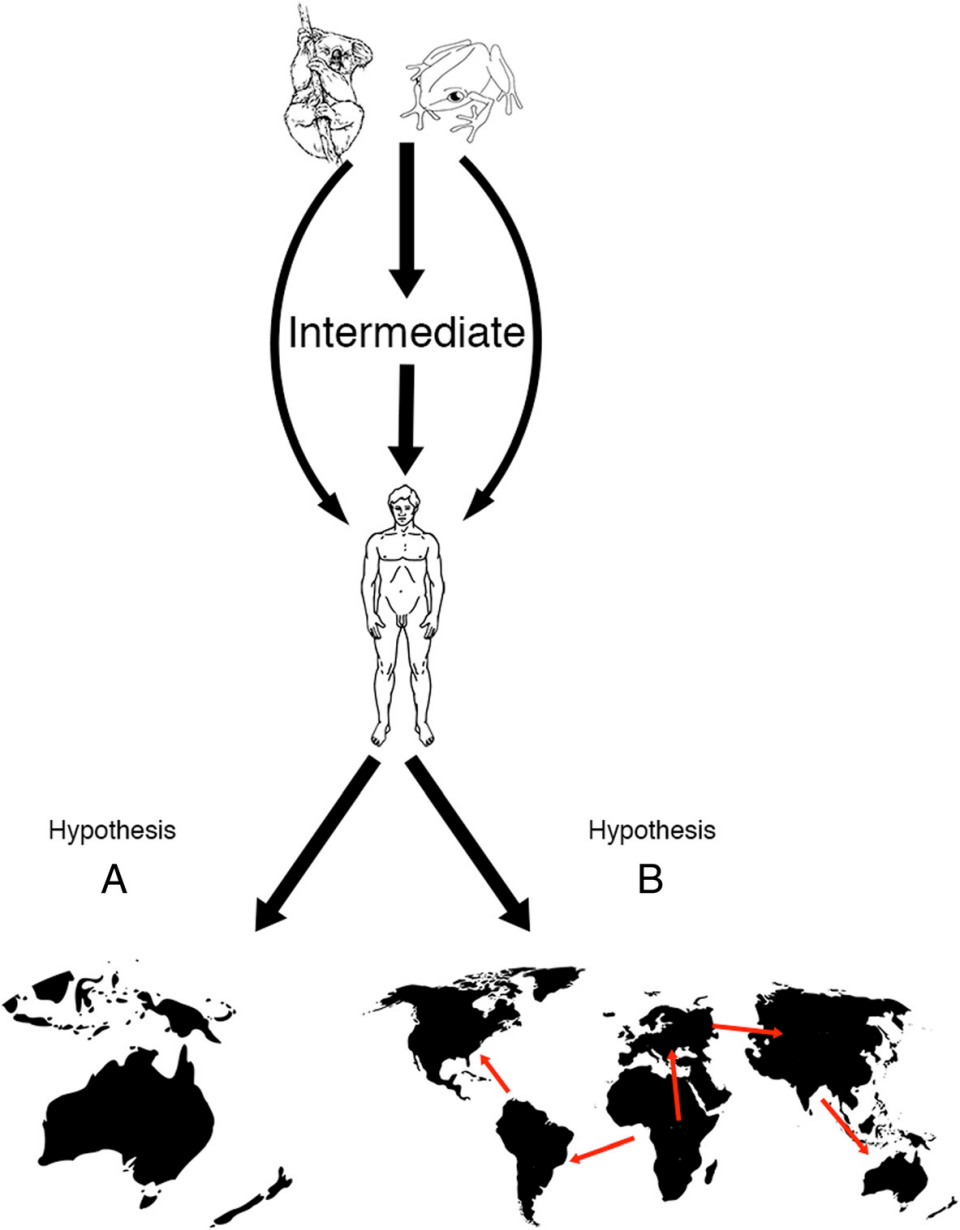
Evolutionary hypothesis model describing two alternate hypotheses for the characteristic deep-branching of the Australian indigenous strains SH511 and 1979. In hypothesis A, Australian indigenous strains evolved from a separate zoonotic (or intermediate) transmission event, and continued to evolve in isolation from non-indigenous human *C. pneumoniae* strains. In hypothesis B, all human *C. pneumoniae* strains disseminated from a single zoonotic (or intermediate) transmission event and evolved separately in response to differing ecological functions

Given this new evidence and our previous data suggesting that *C. pneumoniae* strains in humans likely originated from a zoonotic event(s) [9, 23], it is interesting to speculate on the origin of these indigenous human *C. pneumoniae* strains. Two possible evolutionary hypotheses to explain the deep-branching of these strains are proposed: (A), the Australian indigenous strains have evolved from a separate zoonotic transmission event, or alternate intermediate strain, to that of the other human *C. pneumoniae* strains. These ancestral strains were subsequently endemic on the Australian continent and continued to evolve in isolation to the non-indigenous *C. pneumoniae* strains. Alternatively (B), all human *C. pneumoniae* strains disseminated from a common intermediate strain, resultant from a single zoonotic event several thousand years ago, and evolved separately in response to their different ecological niches (Fig. 7). Our findings provide support for both hypotheses.

With respect to hypothesis (A), estimations from both BEAST and ClonalFrame analyses indicate an MRCA for the indigenous strains several hundred years prior to the first reported visitation of the Australian continent by Dutch or British explorers [42, 43]. This suggests the possibility that an endemic strain similar to our strains may have been circulating within the indigenous population prior to the arrival of European colonisation. Given the sequence similarity of the indigenous strains to the koala and bandicoot *C. pneumoniae* strains at several key loci (the absence of *guaA*-*add*, polymorphisms in *pmpE/F4* and the *IncA* gene expansion), as well as those previously described [23], it is possible that a strain similar to these animal strains was zoonotically transmitted to humans on the Australian continent. Hunter-gatherer communities lived in close proximity and interacted with wild animals throughout human history, which would facilitate the transmission of a pathogen to humans. Serological studies examining the prevalence of chlamydial infection in remote indigenous communities have reported levels of almost 60 % adult female seroprevalence to *C. pneumoniae* [24]. Several species of native Australian marsupials [9, 23, 37, 44] as well as an amphibian [7, 23] have been demonstrated to have genetic sequence similar to that of the koala LPCoLN strain. Studies have shown that koala and bandicoot *C. pneumoniae* strains readily infect various human-derived cell lines [3, 45, 46], and evidence for human carotid artery and PBMC strains which are genotypically similar to the koala strain at the *ompA* and *yge-urk* intergenic spacer loci have been reported [47]. If the distinct phylogenetic clustering of SH511 and 1979 is a result of a separate zoonotic event to that of the main human *C. pneumoniae* lineage, then it is likely that the animal strain that they have evolved from is still unknown, and probably more similar to the frog DC9 strain in sequence and nucleotide content.

The alternate hypothesis (B), is that all human *C. pneumoniae* strains disseminated from a single zoonotic event (presumably in Americas or Europe) and then differentiated along separate evolutionary paths, dependent on their geographical and disease niche. The estimated MRCA for indigenous and non-indigenous human strains differs by less than 200 years, whilst their phylogenetic distance is significantly closer, compared to the animal strains. The overall nucleotide pairwise identity of the Australian indigenous strains is more similar to other human strains of *C. pneumoniae*, even when significant similarities to animal strains at discrete loci are included. There are two possible mechanisms to explain the dissemination of these particular strains: Firstly - various strains of *C. pneumoniae* were circulating in the worldwide human population approximately 40 thousand years ago, which is well prior to the colonisation of the Australian continent [48], and that one or some of these strains came to the continent with the arrival of the indigenous peoples. This would account for the characteristic sequence polymorphisms present in the SH511 and 1979 but not in other human *C. pneumoniae* strains. Alternately - the worldwide variation in human *C. pneumoniae* is far greater than has yet been determined, and several strain types were introduced to the Australian continent with European colonisation. This in turn accounts for the overall sequence similarity of the SH511 and 1979 strains to non-indigenous human *C. pneumoniae* strains, in particular WA97001, with which it shares a considerable number of SNPs, as opposed to the Australian marsupial strains, LPCoLN and B21. In both cases, genetically distinct subpopulations of *C. pneumoniae* could have spread throughout, and evolved in isolation within the indigenous Australian population. Genotypic variation amongst concurrent populations of monomorphic bacteria resulting from selective sweeps is well documented in both *Chlamydia* [49, 50] and other bacterial species [51]. The differentiation of the main human *C. pneumoniae* lineage from both the indigenous and animal lineages could be explained by adaptation of these strains to selective and antigenic pressure as a result of extensive antibiotic treatment regimes [52].

Whilst our study provides evidence for a phylogenetically and genetically distinct branch of human *C. pneumoniae*, these inferences are made on a relatively small sample size, taken from two individuals from remote communities in the same state, over two decades ago. It is highly unlikely that sampling from the same remote communities and wider ranging communities will uncover the same strains as documented in this study; given the increased interaction between members of remote indigenous communities and neighbouring townships, as well as expanded antibiotic treatment regimes for a range of bacterial infections, including *Chlamydia*, within these communities. It is also possible that greater sampling for *C. pneumoniae* in countries outside Australia would uncover a wider range of strains, some of which may be similar to those described in this study.

## Conclusion

In summary, we used a combination of comparative genomic and phylogenetic methods to determine the evolutionary position of three Australian human *C. pneumoniae* strains within the greater *C. pneumoniae* tree. Our study demonstrated a phylogenetically distinct human *C. pneumoniae* clade consisting of two Australian indigenous strains, that branched earlier in the human *C. pneumoniae* evolutionary tree with an estimated MRCA predating the exploration and colonisation of the continent by European settlers by several hundred years. Our findings indicate that a unique strain of *C. pneumoniae* evolved in isolation within the Australian indigenous population, as evidenced by the unique recombination profiles and distinct sequence polymorphisms in these strains. This suggests that a far greater level of sequence diversity is present amongst human and animal *C. pneumoniae* strains than previously surmised, and that further sampling of *C. pneumoniae* isolates from wider geographical regions may uncover strains which have evolved similarly to this unique *C. pneumoniae* clade.

## Methods

### Description of *Chlamydia pneumoniae* strains, cell culturing and DNA purification

Three Australian *C. pneumoniae* cultured isolates (WA97001, SH511 and 1979) were used for comparative analyses in this study. The non-indigenous isolate WA97001 is a clinical nasopharyngeal isolate from Western Australia [26] whilst isolates SH511 and 1979 are indigenous Australian isolates from two separate patients in remote Northern Territory communities [24, 25].

Isolate WA97001 was propagated on McCoy cells in T75 flasks for five passages, based on a previously described method [46]. Infected cells were pooled and semi-purified using a sonication and centrifugation method prior to passage. The final semi-purified product was stored in an equal volume of SPG media [53]. 500 μl of this semi-purified material was used for DNA extraction. Isolates SH511 and 1979 were extracted from non-viable archival culture material [23]; 500 μl of each isolate was used for DNA extraction.

DNA extraction was performed using phenol:chloroform:IAA, based on a well described method [54], with the addition of 2 μl of glycogen prior to ethanol precipitation at −20 °C overnight. Precipitated DNA was dissolved in 50 μl of TE buffer. 500 ng of extracted DNA was used to perform pan-*Chlamydiales* 16S *rRNA* [55] and *C. pneumoniae* specific *RpoB* [56] PCR to confirm the presence of *C. pneumoniae* DNA, and 500 ng of stock DNA was electrophoresed on a 0.8 % TBE agarose gel to confirm high molecular weight DNA. Each DNA extraction yielded greater than 2 μg of high molecular weight genomic DNA, which was used for sequence capture and Illumina HiSeq 2500 whole genome sequencing at the Institute for Genome Sciences, Baltimore, Maryland.

### Sequence capture, whole genome sequencing and assembly

Sequence capture was performed on total DNA extracted from WA97001, SH511 and 1979 with Agilent SureSelect^XT^ DNA capture probes designed to *C. pneumoniae* reference strain AR39, using a hybridisation capture and amplification process [27–29]. Captured and amplified products were sequenced using the Illumina HiSeq 2500 platform, resulting in paired-end 100 base pair reads. Read quality was checked with FastQC (http://www.bioinformatics.bbsrc.ac.uk/projects/fastqc/) and genomes were assembled de novo using SPAdes 3.0.0 with SPAdes 3.0.0 with k-mer values set to of 15, 21, 33, 51 and 71 [57]. All assembled contigs were aligned to the reference *C. pneumoniae* AR39 genome using BLASTn to remove non-chlamydial contigs. Concatenated genome contigs were annotated using the RAST pipeline [58] and manually curated using ARTEMIS [59]. Total read depth of WA97001, SH511 and 1979 was calculated by mapping the raw reads to complete genome of *C. pneumoniae* AR39 using the BWA-backtrack algorithm with BWA aligner [60]. Raw reads were also mapped to the complete genome of *C. pneumoniae* LPCoLN for comparison. The BWA parameters used include the number of differences allowed between the reference and query set at 0.04 and the number of differences allowed in the seed was 2. The maximum number of gaps allowed in the alignment was 1 and the gap penalty was set at 11.

### Phylogenetic and recombination analyses

*De novo* assemblies and readmapped assembled consensus sequences for WA97001, SH511 and 1979 were aligned to the existing human *C. pneumoniae* whole genome sequences [10, 19–22, 61] and animal *C. pneumoniae* strains LPCoLN, B21 and DC9 [9, 22, 37] in Geneious 6.1.8 [62] using the MAFFT plugin implementation [63]. Coverage analyses for readmapped assemblies and manual curation of annotated genomes was performed using ARTEMIS [59].

Phylogenetic analyses were performed on whole genome alignments, with the LPCoLN koala [9] *C. pneumoniae* strain indicated as an outlier. Whole genome alignments were also filtered for poorly aligned and gap regions using Gblocks 0.91b [64]. Mid-point rooted trees were constructed with the MrBayes plugin [65] in Geneious, utilising a Jukes-Cantor substitution model with with four Markov Chain Monte Carlo (MCMC) chains and 1.1 million cycles, sampled every 1000 generations and the first 10,000 trees discarded as burn-in. Estimates of strain evolution over time were performed on whole genome alignments using the BEAST package [31]. Indigenous, non-indigenous and animal isolates were defined in separate taxon sets and a GTR nucleotide substitution model was employed. MRCA priors were set at a normal distribution with a mean of 95.2 +/− 7.4 [66]. MCMC chain length was set to 5 × 10^7^ to ensure effective sample sizes were sufficient for strong posterior distribution statistics. ClonalFrame [67] was used to determine homologous recombination within *C. pneumoniae* genomes, and progressive MAUVE [68] was used to generate the input alignments. Three successive runs of ClonalFrame were performed on the whole genome alignment, each with 20,000 iterations and 10,000 of these discarded as burn-in. The three runs were checked for convergence and their trees combined for analysis. An additional Phi test for recombination was performed in SplitsTree4 [34] using the whole genome alignment generated by MAFFT in Geneious.

The accession numbers for the *C. pneumoniae* whole genome sequences used in the comparative analyses and phylogenies are outlined in Table 2.

**Table 2:** *C. pneumoniae* strain designations and accession numbers

Strain designation	Accession number	Reference/s
A03	SRP056807	[15, 21]
AR39	NC_002179.2	[19]
B21	NZ_AZNB01000000	[37]
CM1	ERS640705	[22]
CV14	ERS640706	[22]
CV15	ERS640707	[22]
CWL011	ERS640708	[22]
CWL029	NC_000922.1	[10]
DC9	ERS640710	[22]
GiD	ERS640711	[22]
H12	ERS640712	[22]
IOL207	-	[22]
J138	NC_002491.1	[20]
K7	ERS640713	[22]
LPCoLN	NC_017285.1	[9]
MUL2216	ERS640714	[22]
Panola	ERS640715	[22]
PB1	ERS640716	[22]
PB2	ERS640717	[22]
SH511	SRP061961	[25] This study
TOR-1	SRP056806	[16, 21]
TW183	NC_005043.1	Unpublished
U1271	ERS640718	[22]
UZG1	ERS640719	[22]
WA97001	SRP062032	[26] This study
Wien1	ERS640720	[22]
Wien2	ERS640721	[22]
Wien3	ERS640722	[22]
YK41	ERS640723	[22]
1979	SRP062031	[25] This study

### Description of polymorphic hotspots in *C. pneumoniae* whole genome alignments

*De novo* and readmapping assemblies were used to construct whole genome alignments with previously described human and animal *C. pneumoniae* whole genomes in MAFFT [63] in Geneious [62]. Single nucleotide polymorphisms (SNPs) and insertions/deletions were detected using the Variations/SNPs tool in Geneious, and larger scale differences were detected via manual scanning of the genome alignment. Sequence for genes which appeared to have significant deletions or insertions were manually extracted and sequence run against the BLAST [69] database to determine closest homologs. Sequences were translated and searched against the SMART database [70] to predict any changes in functional domains or protein motifs.

### Availabilty of supporting data

The WA97001, SH511 and 1979 whole genome sequencing projects can be found on National Center for Biotechnology Information (NCBI) BioProject under accession numbers [Bioproject:PRJNA291806, Bioproject:PRJNA291802 and Bioproject:PRJNA291805] with reads deposited in the Short Reads Archive under accession numbers [SRA:SRR2144962, SRA:SRR2144961 and SRA:SRR2144960] respectively.

### Ethics statement

This study was approved by the ethics committee of the Queensland University of Technology and Menzies School of Health Research, Human Research Ethics Committee. Ethics approval for the collection and analysis of strains SH511, 1979 and WA97001 were obtained from Queensland University of Technology, Menzies School of Health Research and the Princess Margaret Hospital for Children.

## Abbreviations

bp: base pair
*pmp*: polymorphic membrane protein
*inc*: inclusion protein
PCR: polymerase chain reaction
MRCA: most recent common ancestor
SNP: single nucleotide polymorphism
*gua*A: guanine monophosphate synthetase
*gua*B: inosine-5-monophosphate dehydrogenase
*add*: adenosine deaminase
AA: amino acid
